# Apigenin as a Multi-Targeted Agent in Gastrointestinal Cancers: A Systems Pharmacology Approach

**DOI:** 10.34172/apb.025.45970

**Published:** 2025-10-11

**Authors:** Duaa Kamel Al-Moussawi, Aseel Kamil Mohammed Al-Mosawi, Awatif Mokar Dayesh, Mohammad Hosseinzadeh Hesari, Hamid Cheshomi

**Affiliations:** ^1^General Directorate of Education in Thi-Qar, Ministry of Education, Nasiriyah, Iraq; ^2^Department of Biology, Faculty of Science, University of Thi Qar, Nasiriyah, Iraq; ^3^Non-Communicable Diseases Research Center, Sabzevar University of Medical Sciences, Sabzevar, Iran; ^4^Department of Operating Room, Faculty of Paramedicals, Sabzevar University of Medical Sciences, Sabzevar, Iran; ^5^Razavi Khorasan Agricultural and Natural Resources Research and Education Center, AREEO, Mashhad, Iran; ^6^Cellular and Molecular Research Center, Sabzevar University of Medical Sciences, Sabzevar, Iran

**Keywords:** Apigenin, Gastrointestinal cancer systems pharmacology, Gene network, Survival analysis, Molecular docking

## Abstract

**Introduction:**

Apigenin, a dietary flavonoid found in various medicinal plants, has demonstrated notable anticancer effects. However, its multi-targeted molecular mechanisms in gastrointestinal (GI) cancers remain poorly understood. This study aimed to comprehensively identify the key molecular targets and signaling pathways influenced by apigenin in five major GI cancers: esophageal, gastric, colorectal, pancreatic, and liver cancers.

**Methods:**

Natural plant sources of apigenin were identified, and apigenin-related genes were extracted from public databases and scientific literature. Protein–protein interaction (PPI) networks were constructed, followed by hub gene identification using CytoHubba software. Functional enrichment analyses, survival analysis, and gene expression profiling were conducted using the STRING, DAVID, and GEPIA platforms. Molecular docking was performed to evaluate the binding affinities between apigenin and key oncogenic proteins.

**Results:**

Hub genes, including TP53, AKT1, STAT3, BCL2, and HIF1A, were identified as central nodes in PPI network. Expression and survival analyses revealed that HIF1A, IL6, and STAT3 were significantly upregulated in tumors and correlated with poorer prognosis. Enrichment analyses indicated that apigenin-responsive targets were significantly involved in the PI3K-Akt, MAPK, JAK-STAT, and mTOR signaling pathways. Docking studies confirmed the high binding affinity of apigenin to key targets, such as AKT1 and PTEN.

**Conclusion:**

The results suggest that apigenin exerts its anticancer effects by modulating multiple oncogenic pathways and interacting with key regulatory proteins involved in tumor progression and survival. This integrative systems pharmacology study provides suggestive evidence for the pleiotropic anticancer potential of apigenin in GI cancers and supports its development as a multi-target agent in precision oncology.

## Introduction

 Gastrointestinal (GI) cancers represent a significant global health burden, encompassing malignancies of the esophagus, stomach, small intestine, colon, rectum, and anus.^1,2^ These cancers collectively exhibit high incidence and mortality rates, posing substantial challenges for early diagnosis and effective treatment.^3^ Despite advances in surgical techniques, chemotherapy, and radiotherapy, many patients with GI cancers continue to experience poor outcomes due to factors such as late-stage diagnosis, metastasis, and the development of drug resistance.^4^ The complex interplay of genetic, environmental, and lifestyle factors in the pathogenesis of these malignancies underscores the need for more comprehensive and targeted therapeutic strategies.^5^ The global incidence of GI cancers is rising, with millions of new cases diagnosed annually, contributing to a significant proportion of cancer-related deaths worldwide.^6^

 Among GI malignancies, individual cancer types exhibit distinct epidemiological patterns and burdens. Globally, in 2020, esophageal cancer (EC) accounted for an estimated 604,100 new cases and 544,100 deaths, ranking as the seventh most commonly diagnosed cancer and the sixth leading cause of cancer-related mortality worldwide. The highest incidence and mortality rates were observed in Eastern Asia and Sub-Saharan Africa.^7^ In 2022, gastric cancer (GC) accounted for approximately 1 million new cases (4.9% of all cancers) and was the fifth leading cause of cancer mortality.^8^ Incidence of GC varies significantly by geographic region.^9^ Additionally, colorectal cancer (CRC) represents approximately 10.0% of all cancer cases and 9.0% of cancer-related deaths worldwide.^10^ Moreover, pancreatic cancer (PC) remains one of the most lethal malignancies, with a five-year survival rate of less than 12%, primarily due to late diagnosis, aggressive progression, and resistance to standard therapies.^11^ In 2020, liver cancer (LC), predominantly hepatocellular carcinoma (HCC), accounted for approximately 905,700 new cases and 830,200 deaths globally, making it the sixth most frequently diagnosed cancer and the fourth leading cause of cancer-related deaths. Without effective prevention, the burden is expected to rise to 1.4 million cases and 1.3 million deaths by 2040.^12^ These persistently high mortality rates across GI cancers underscorethe critical need for improved diagnostic tools and more effective, targeted therapeutic strategies.

 Natural compounds have emerged as promising candidates for cancer therapy, offering potential avenues for both prevention and treatment across various malignancies.^13,14^ Among these, flavonoids, a diverse group of plant-derived polyphenols, have attracted considerable attention due to their broad biological activities and relatively low toxicity.^15,16^ Apigenin (4′,5,7-trihydroxyflavone), a naturally occurring flavonoid found in many fruits, vegetables, and herbs, has been extensively studied for its multifaceted anticancer effects.^2,16^ These effects include modulation of multiple cell signaling pathways, induction of apoptosis, inhibition of cell proliferation, and regulation of the tumor microenvironment.^1^ Due to its favorable bioavailability and mechanistic versatility, apigenin has been proposed as a promising dietary supplement or adjuvant chemotherapeutic agent.^17^ Given the rising global incidence of GI cancers and the current limitations of treatment options, apigenin and related natural compounds have garnered significant interest as potential candidates for novel therapeutic strategies.^18^

 To fully elucidate the mechanisms of action of anticancer compounds, particularly in the context of GI malignancies, a shift from traditional reductionist approaches to integrative strategies such as systems biology and systems pharmacology is essential.^19,20^ These approaches provide comprehensive frameworks for studying complex biological networks involving genetic, environmental, and lifestyle factors, all of which play pivotal roles in the onset and progression of GI cancers. Systems biology enables researchers to analyze the intricate interactions among genes, proteins, metabolites, and their environmental contexts, thereby offering insights into cellular phenotypes and drug responses.^21,22^ Building on this foundation, systems pharmacology integrates pharmacological data with systems-level models to understand the effects of drugs across multiple biological scales.^23^ Using these integrative tools, scientists can identify key molecular targets, predict drug responses, and devise personalized treatment regimens.^24^ Additionally, these methods provide valuable insights into the mechanisms of drug resistance and toxicity, supporting the development of safer and more effective therapies.^25^ This approach is particularly relevant in the study of natural products such as apigenin, where a multitude of potential targets necessitates systems-level strategies to decode their full therapeutic potential.^26^

 This study was designed to elucidate the key molecular mechanisms through which apigenin exerts its anticancer effects in GI malignancies using an integrative systems biology and pharmacology framework. Specifically, it focused on five major GI cancers, each contributing significantly to the global cancer burden and presenting unique challenges in diagnosis and treatment. Apigenin-responsive genes and proteins implicated in these cancers were identified through rigorous literature mining and database integration methods. Subsequently, interaction networks were constructed to uncover molecular interconnections, followed by functional enrichment analyses to identify the most relevant biological processes and signaling pathways involved. Hub genes were determined through topological network analysis to identify central regulatory elements. Finally, molecular docking simulations were conducted to evaluate the potential binding interactions between apigenin and the identified hub proteins. Despite extensive research on apigenin as an anticancer agent, its multi-targeted effects across different GI cancers remain incompletely understood. Most existing studies focus on individual signaling pathways or single cancer types, leaving a significant gap in understanding the integrated network-level mechanisms across esophageal, gastric, colorectal, liver, and pancreatic cancers. To address this gap, the present study employs a systems pharmacology approach to systematically identify and compare the molecular targets, signaling networks, and preclinical therapeutic potential of apigenin across these malignancies. By providing the first comprehensive, systems-level comparison of apigenin’s effects in major GI cancers, this work aims to highlight key hub genes, pathways, and potential combination strategies, thereby guiding future translational and preclinical research.

## Methods

###  Databases and Software Tools Used

 This study utilized a range of well-established bioinformatics databases and computational software to ensure the accuracy and reproducibility of the analyses. The following software platforms were employed for computational modeling and visualization: AutoDock Vina (v1.5.6) for molecular docking simulations; Cytoscape (v3.10.3) for network visualization and hub gene analysis using the CytoHubba plugin; Discovery Studio Visualizer (Biovia, San Diego, CA) for protein–ligand interaction mapping; Gaussian 09 for molecular geometry optimization; and Open Babel for molecular structure conversion and partial charge assignment. These tools collectively facilitated multi-level analyses, including spanning phytochemical screening, gene–protein interaction networks, differential gene expression, survival correlations, and structural docking of apigenin to its molecular targets. Additionally, several specialized online databases were utilized for various analyses throughout the study (Table 1).

**Table 1:** Online databases used in this study

**No.**	**Database Name**	**Abbreviation**	**Web Address**
1	Binding Database	BindingDB	https://www.bindingdb.org
2	Database for Annotation, Visualization and Integrated Discovery	DAVID	https://david.ncifcrf.gov
3	DrugBank	N/A	https://go.drugbank.com
4	GeneCards	N/A	https://www.genecards.org
5	Gene Expression Profiling Interactive Analysis	GEPIA	http://gepia.cancer-pku.cn
6	Gene Multiple Association Network Integration Algorithm	GeneMANIA	https://genemania.org
7	Phytochemical and Ethnobotanical Databases	N/A	https://phytochem.nal.usda.gov
8	Pharmacogenomics Knowledgebase	PharmGKB	https://www.pharmgkb.org
9	Protein Data Bank	PDB	https://www.rcsb.org
10	Search Tool for Interactions of Chemicals and Targets.	STITCH	http://stitch.embl.de
11	Search Tool for the Retrieval of Interacting Genes/Proteins	STRING	https://string-db.org
12	Therapeutic Target Database	TTD	http://db.idrblab.net/ttd

###  Identification of Natural Sources Rich in Apigenin

 To identify natural plant sources rich in apigenin, we explored Dr. Duke’s Phytochemical and Ethnobotanical Databases (https://phytochem.nal.usda.gov), a publicly available resource maintained by the U.S. Department of Agriculture (USDA) that compiles detailed phytochemical data from peer-reviewed studies.^27^ Using a keyword-based search for “apigenin,” we retrieved a list of plant species and evaluated them based on the reported quantifiable concentrations of apigenin in various plant parts, such as leaves, flowers, and seeds. These findings highlight potential plant candidates that could serve as valuable dietary or pharmacological sources of apigenin for future translational applications.

###  Collection of Apigenin-Associated Targets in GI Cancers

 To identify genes and proteins associated with GI cancers that respond to apigenin, we conducted a systematic literature search in the PubMed database (NCBI), focusing on experimentally validated findings in esophageal, gastric, colorectal, pancreatic, and liver malignancies. The search strategy combined controlled vocabulary (MeSH terms) and free-text keywords, including: “Apigenin” OR “4’,5,7-trihydroxyflavone” AND “Esophageal cancer” OR “Esophageal carcinoma” OR “Gastric cancer” OR “Stomach neoplasm” OR “Colorectal cancer” OR “Colon carcinoma” OR “Rectal neoplasm” OR “Pancreatic cancer” OR “Pancreatic carcinoma” OR “Liver cancer” OR “Hepatocellular carcinoma” OR “HCC”. Boolean operators (AND/OR) were applied to optimize specificity and sensitivity. Only peer-reviewed original research articles reporting experimentally validated apigenin-responsive genes or proteins were included, while computational predictions, reviews, case reports, and studies unrelated to GI cancers were excluded.

 In parallel, high-confidence molecular targets of apigenin were compiled from several curated pharmacological and interaction databases, including DrugBank, PharmGKB, STITCH, TTD, and BindingDB. Interactions from STITCH were filtered to include only those with confidence scores ≥ 0.7, and BindingDB entries were restricted to those with Kd values less than 10 μM, ensuring biological relevance and reliability.

 Furthermore, to facilitatedata integration and ensurecross-platform consistency, each gene was annotated with standardized identifiers, including HGNC symbols, Entrez Gene IDs, Ensembl IDs, OMIM entries, and UniProtKB accessions obtained throughGeneCards.

###  Network Construction and Functional Enrichment Analysis

 To investigatethe functional roles and interaction networks of apigenin-modulated genes in GI cancers, we employed a two-step approach involvingnetwork construction and functional enrichment analysis. First, gene and protein interaction networks were constructed using STRING (https://string-db.org),^28^ which integrates known and predicted interactions based on experimental data, curated databases, and computational predictions. Additionally, GeneMANIA (https://genemania.org)^29^ was utilized to incorporate further context-specific interactions, including co-expression, pathway co-membership, and protein domain similarity. These networks enabledvisualization of functional gene associations and facilitatedclustering analysis.

 Next, functional enrichment analysis was performedto identify significantly overrepresented Gene Ontology (GO) categories (biological processes [BP], molecular functions [MF], and cellular components [CC]), as well asKEGG pathways. For this purpose, STRING’s built-in enrichment tool and DAVID (https://david.ncifcrf.gov)^30^ were used. To control for multiple comparisons and reduce false-positive findings, all p-values were adjusted using the Benjamini–Hochberg false discovery rate (FDR) procedure. Terms with q-values < 0.05 were considered statistically significant.

 This integrative approach offers mechanistic insights into how apigenin influences cancer-related pathways and highlights key biological processes involved in GI tumorigenesis, with robust results that account for multiple testing corrections.

###  Identification of Hub Genes Using CytoHubba

 To identify key regulatory genes within the apigenin-responsive gene network in GI cancers, hub gene analysis was performed using Cytoscape (version 3.10.3), an open-source platform for visualizing and analyzing molecular interaction networks. Protein–protein interaction (PPI) data retrieved from STRING were imported into Cytoscape, and the CytoHubba plugin^31^ was used to evaluate network topology and rank genes based on centrality metrics.

 Among the available algorithms, the degree method, which ranks nodes based on the number of direct interactions, was selected to identify highly connected genes, reflecting their potential significance in maintaining network integrity and biological function. The top ten genes with the highest degree values were designated as hub genes, as they are presumed to play pivotal roles in the molecular regulatory landscape influenced by apigenin in GI malignancies.

###  Survival Analysis Based on Hub Gene Expression Using GEPIA

 To assess the prognostic significance of the identified hub genes in GI cancers, survival analysis was performed using the GEPIA web server (http://gepia.cancer-pku.cn), an interactive online platform that integrates RNA sequencing expression data from The Cancer Genome Atlas (TCGA) and the Genotype-Tissue Expression (GTEx) project.^32^

 Overall survival (OS) curves were generated for each hub gene by stratifying patients into high- and low-expression groups using the median gene expression value as the threshold. The Kaplan–Meier method was employed to estimate survival distributions, and log-rank tests were conducted to assess statistical significance, with *P* < 0.05 considered to be significant. Hazard ratios (HRs) and 95% confidence intervals (CIs) were calculated and displayed on the survival plots to indicate the relative risk associated with differential gene expression.

###  Comparative Expression Analysis of Hub Genes in Normal and Tumor Tissues of GI Organs

 To evaluate the differential expression patterns of the identified hub genes between normalandtumortissues across major gastrointestinal organs, including the esophagus, stomach, colorectum, liver, and pancreas, transcriptomic profiling was conducted using the GEPIA web server (http://gepia.cancer-pku.cn). GEPIA provides standardized RNA sequencing data derived from The Cancer Genome Atlas (TCGA) and Genotype-Tissue Expression (GTEx) projects, enabling robust comparative analyses across tissue types.

 Gene expression values were measured in transcripts per million (TPM)and visualized using boxplots generated by the GEPIA differential expression analysis module. For each organ, tumor tissues were compared with their corresponding normal tissues. Statistical significance was assessed using Student’s t-test, applyingdefault thresholds of |log₂ fold change (FC)| ≥ 1 and *P* < 0.01. This analysis facilitated the identification of organ-specific expression profiles and underscored the diagnostic and prognostic potential of hub genes in GI cancers.

###  Structure-Based Molecular Docking of Apigenin with Target Proteins

 To elucidate the molecular interactions between apigenin and the selected protein targets, structure-based molecular docking was performed using AutoDock Vina.^33^ The high-resolution three-dimensional (3D) structures of the ten target proteins were retrieved from the Protein Data Bank (PDB). Prior to docking, the protein structures were preprocessed by removing crystallographic water molecules, ligands, and cofactors to prevent steric clashes and artifacts in the binding interactions.^34^ Hydrogen atoms were added, and protonation states were adjusted to reflect physiological pH (7.4) for accurate electrostatic modeling.^1^

 The 3D structure of apigenin (PubChem CID: 5280443; molecular formula: C₁₅H₁₀O₅) was obtained from PubChem and geometry-optimized using Gaussian 09.^35^ Molecular structure conversion and optimization were performed using Open Babel,^36^ and partial atomic charges were assigned using the Gasteiger method to ensure realistic electrostatic interactions.^37^

 Docking simulations were performed using AutoDock Vina, which employs a stochastic global optimization algorithm to predict optimal binding poses.^38^ A grid box was centered on the predicted binding site with a grid spacing of 0.375 Å to allow exhaustive conformational sampling. For each protein–ligand pair, 20 docking poses were generated and ranked based on binding affinity using Vina’s empirical scoring function, which incorporates hydrogen bonding, van der Waals interactions, and electrostatics.^39^ The pose with the lowest binding energy was selected for further visualization and interpretation.

 To visualize key ligand–protein interactions, BIOVIA Discovery Studio visualizer (BIOVIA, San Diego, CA) was used to generate 2D interaction diagrams. These visualizations highlighted the hydrogen bonds and hydrophobic contacts between apigenin and critical amino acid residues in the binding site^40-42^, providing insights into the structural determinants of binding affinity and specificity.

## Results and Discussion

###  Natural Plant Sources and Bioavailability

 Apigenin is a naturally occurring flavonoid found abundantlyin a wide variety of medicinal and dietary plants. Its concentration varies acrossspecific anatomical structures, such as leaves, stems, flowers, fruits, and roots, depending on the plant species. Identifying and cataloging these botanical sources is crucial not only for understanding the phytochemical distribution of apigenin but also for enabling its efficient extraction and targeted therapeutic application. Table 2 provides a curated list of major apigenin-containing plant species, along with the predominant plant parts whereapigenin is most frequently detected. This dataset serves as a foundational reference for future pharmacognostic studiesand the development of apigenin-enriched therapeutic agents, particularly in the context of GI cancers.

**Table 2:** Herbal resources of apigenin

	**Plant Name**	**Plant Part**		**Plant Name**	**Plant Part**
1	*Achillea millefolium*	Plant	52	*Acinos suaveolens*	Shoot
2	*Apium graveolens*	Plant	53	*Medicago sativa*	Shoot
3	*Artemisia dracunculus*	Plant	54	*Cynara cardunculus*	Leaf
4	*Camellia sinensis*	Leaf	55	*Stellaria media*	Shoot
5	*Centaurea calcitrapa*	Plant	56	*Ephedra sinica*	Shoot
6	*Chamaemelum nobile*	Plant	57	*Centaurea cyanus*	Shoot
7	*Colchicum autumnale*	Tuber	58	*Ginkgo biloba*	Pollen/Spore
8	*Conyza canadensis*	Plant	59	*Crataegus laevigata*	Leaf
9	*Coriandrum sativum*	Fruit	60	*Crataegus laevigata*	Bud
10	*Daphne genkwa*	Flower	61	*Marrubium vulgare*	Shoot
11	*Daucus carota*	Fruit	62	*Marrubium vulgare*	Leaf
12	*Digitalis purpurea*	Flower	63	*Salix alba*	Leaf
13	*Echinacea spp*	Leaf	64	*Stachys officinalis*	Shoot
14	*Ginkgo biloba*	Leaf	65	*Serenoa repens*	Fruit
15	*Glechoma hederacea*	Plant	66	*Echinacea angustifolia*	Leaf
16	*Glycyrrhiza glabra*	Root	67	*Equisetum arvense*	Plant
17	*Hydnocarpus wightiana*	Seed	68	*Euphrasia officinalis*	Plant
18	*Jatropha gossypifolia*	Leaf	69	*Matricaria recutita*	Flower
19	*Linum usitatissimum*	Plant	70	*Apium graveolens*	Seed
20	*Lycopodium clavatum*	Plant	71	*Crataegus monogyna*	Fruit
21	*Marrubium vulgare*	Plant	72	*Crataegus rhipidophylla*	Fruit
22	*Matricaria recutita*	Plant	73	*Crataegus rhipidophylla*	Leaf
23	*Mentha aquatica*	Plant	74	*Crataegus rhipidophylla*	Bud
24	*Mentha spicata*	Leaf	75	*Cichorium intybus*	Shoot
25	*Mentha x rotundifolia*	Shoot	76	*Trigonella foenum -graecum*	Plant
26	*Ocimum basilicum*	Plant	77	*Juniperus communis*	Plant
27	*Olea europaea*	Leaf	78	*Pimpinella anisum*	Seed
28	*Passiflora incarnata*	Plant	79	*Pueraria montana*	Root
29	*Perilla frutescens*	Seed	80	*Scutellaria lateriflora*	Plant
30	*Petroselinum crispum*	Plant	81	*Scutellaria baicalensis*	Leaf
31	*Phaseolus vulgaris*	Plant	82	*Maclura pomifera*	Fruit
32	*Phoenix dactylifera*	Stem	83	*Maclura pomifera*	Tissue Culture
33	*Plantago major*	Leaf	84	*Eucalyptus globulus*	Leaf
34	*Pogostemon cablin*	Plant	85	*Anethum graveolens*	Leaf
35	*Prosopis juliflora*	Plant	86	*Origanum vulgare*	Leaf
36	*Prunus cerasus*	Plant	87	*Petroselinum crispum*	Tissue Culture
37	*Rosmarinus officinalis*	Plant	88	*Salvia officinalis*	Shoot
38	*Salix alba*	Bark	89	*Satureja montana*	Plant
39	*Salvia officinalis*	Plant	90	*Satureja montana*	Shoot
40	*Scutellaria galericulata*	Plant	91	*Malus domestica*	Heart Wood
41	*Silybum marianum*	Fruit	92	*Crataegus laevigata*	Fruit
42	*Tanacetum vulgare*	Plant	93	*Crataegus monogyna*	Flower
43	*Teucrium polium*	Plant	94	*Crataegus monogyna*	Leaf
44	*Thymus serpyllum*	Plant	95	*Ballota nigra*	Shoot
45	*Thymus vulgaris*	Plant	96	*Agrimonia eupatoria*	Plant
46	*Triticum aestivum*	Seed	97	*Aloysia citrodora*	Plant
47	*Araucaria bidwillii*	Leaf	98	*Ononis spinosa*	Shoot
48	*Anisochilus carnosus*	Plant	99	*Allium sativum var. sativum*	Bulb
49	*Origanum vulgare*	Plant	100	*Artemisia annua*	Shoot
50	*Apium graveolens*	Plant	101	*Ocimum tenuiflorum*	Leaf
51	*Silybum marianum*	Seed	102	*Punica granatum*	Leaf

 As summarized in Table 2, apigenin is present across a diverse range of plant families, with notable concentrations in whole-plant materials, leaves, flowers, shoots, roots, and seeds, and less commonly in specialized structures such as tubers or pollen. Several species, including *Apium graveolens*, *Matricaria recutita*, *Salvia officinalis*, and *Silybum marianum*, are of particular pharmacognostic interest due totheir high apigenin content and traditional use in gastrointestinal and anti-inflammatory herbal remedies. These data provide a botanical framework for targeted extraction strategies and the future development of apigenin-based formulations for GI cancers.

 It should be noted that apigenin presents both opportunities and challenges for experimental and potential clinical applications. Its poor aqueous solubility (0.001–1.63 mg/mL) and limited lipid solubility (~2.16 µg/mL) constrain absorption. However, itsmoderate lipophilicity (log *P* = 2.84), low molecular weight (270.24 g/mol), and low polar surface area enable passive membrane diffusion, including across the blood–brain barrier (BBB).^43^ Studies indicate that apigenin can penetrate the BBB and exert neuromodulatory effects, partly through upregulation of tight junction proteins such as occludin and claudin-5, supporting barrier integrity. Nonetheless, claudin-5, thereby oral bioavailability remains limited due to poor solubility and rapid hepatic metabolism, restricting systemic exposure and potentially reducing clinical efficacy.^44^ These characteristics should be carefully considered when designing *in vivo* studies or developing formulations to enhance delivery and therapeutic impact. Notably,recent advances in apigenin-based nanotechnology have significantly enhanced its therapeutic potential and bioavailability in gastrointestinal and other disease contexts.^45^ For instance, Yang et al. developed hyaluronic acid-coated PLGA nanoparticles (HA-PLGA-API-NPs) for targeted delivery of apigenin to CD44-overexpressing colon cancer cells, demonstrating superior cellular uptake and enhanced in vivo efficacy compared to free apigenin.^46^ Building on this, reported that apigenin-loaded nanoarchitectures effectively overcome drug resistance in colorectal cancer models by improving intracellular drug delivery and bioavailability. Zhao et al. further expanded the scope of apigenin nanodelivery by formulating apigenin-7-glucoside-loaded nanoparticles, which attenuated intestinal ischemia-reperfusion injury via regulation of the ATF3/SLC7A11 pathway and induction of ferroptosis.^47^ Additionally, Sato et al. developed a self-nanoemulsifying drug delivery system (SNEDDS) that markedly enhanced the oral bioavailability of apigenin by promoting transcellular absorption, with improved pharmacokinetic profiles in animal models.^48^ Collectively, these studies highlight the considerable progress in apigenin nanoformulations, emphasizing their potential to translate preclinical efficacy into clinically relevant outcomes.

###  Evidence of Apigenin-Responsive Gene Targets in GI Cancers

 To comprehensively capture the experimental landscape of apigenin-responsive gene regulation in GI cancers, we curated a dataset summarizing the key molecular targets modulated by apigenin across various studies. This compilation includes critical parameters such as cancer type, specific cell lines used, identified genes or proteins, direction of expression change (upregulation or downregulation), experimental methodologies (e.g., qPCR, Western blot, RNA-seq), and relevant references (Table 3).

**Table 3:** Summary of experimentally validated apigenin-responsive genes in GI cancers

**Cancer Type**	**Cell Line(s)**	**Upregulated Targets**	**Downregulated Targets**	**Methods**	**Ref.**
EC	KYSE510, Eca109, KYSE30	Caspase-8, Cleaved PARP, p21, PIG3, p63, p73	IL-6, STAT3, p-STAT3, Cyclin D1, Bcl-2, MMP-9, VEGF, cyclin B1	qRT-PCR, Western blotting, zymography, ELISA, Luciferase reporter assay, *in vivo* xenograft models, Oligonucleotide microarray	^49,50^
GC	AGS, MKN-45, SGC-7901, SNU-638, HGC-27	Bax, p21, p53, Caspase-3, Caspase-9, IκBα, MUC-2, CHOP, ATF4, eIF2α, PERK, ULK1, AMPK, LC3-II, ATG5, Vinculin	Bcl-2, AKT, PI3K, Cyclin D1, MMP-2, MMP-9, IL-1β, IL-8, IL-6, ICAM-1, COX-2, NF-κB, EZH2, HIF-1α, p62, mTOR	Fluorometric assay, Western blotting, qRT-PCR, ELISA	^51-56^
CRC	HCT116, HT29, DLD1, LS174T, SW480, HCT-8, mouse model	p53, APC, p21, LATS1/2, Caspase-3, FADD, Caspase-8, DR5, PARP, c-JNK, HMGB1, Beclin 1, LC3-II, BAX, Caspase-9, FAS	β-catenin, Cyclin D1, YAP/TAZ, Cyclin D1, HSP90AA1, AKT, STAT3, KEAP1, COX-2, Bcl-2, SIRT3, MnSOD, E2F1, E2F3, MMP-9, VEGF, ODC, LATS1, STK3, ERK1, mTOR, NEDD9, PKM2	Western blotting, Immunofluorescence, qRT-PCR, Flow cytometry, Immunoprecipitation, ELISA, Immunohistochemistry, Luciferase reporter assay, High-throughput sequencing, Fluorescent ubiquitination-based cell cycle indicator (FUCCI) assay, MALDI-TOF/TOF analysis, Pull-down assay	^17,51,57-71^
PC	PANC-1, MIA, PaCa-2, BxPC-3	Bax, Caspase-3	GSK-3β, NF-κB, Bcl-2, Cyclin D1, COX-2, VEGF, TNF-α, IL-6, Cyclin D1, HDAC1, Geminin, Cdc6, JAK2/STAT3, HIF-1α, GLUT-1, Src	Western blotting, EMSA, ELISA, qRT-PCR, Flow Cytometry, Fluorometric Assay, Luciferase reporter assay, FUCCI assay	^51,57,67,72-75^
LC	HepG2, Huh7, Hep3B, BEL-7402	p53, Caspase-3, PIG3, ROS LC3/Beclin-1, CPT1A, H3K4me2, PARP, Bax, p21, PTEN	CYP1A1, Bcl-2, STAT3, Survivin, NF-κB, VEGF, DNMT1, CYP1B1, PI3K, Akt, mTOR, KDM1A, SREBP1, FASN, ACC1, Cyclin D1, CDK4, CDK2, Cyclin E, MMP-9, MED28, Nrf2	qRT-PCR, Western blotting, Flow cytometry, ELISA, EMSA, Immunofluorescence, MSP, FUCCI assay, ROS detection assays, Chromatin immunoprecipitation, Immunocytochemistry, Zymography, Microarray analysis	^51,57,64,67,68,76-83^

 The assembled evidence reveals a complex and diverse molecular profile influenced by apigenin, involving genes implicated in critical oncogenic processes such as apoptosis, cell proliferation, inflammation, and metastasis. Notably, several targets, including *TP53*, *BCL2*, and *STAT3, *demonstrated consistent modulation across multiple GI cancer types and cellular models, suggesting their potential role as conserved mediators of apigenin’s therapeutic effects. This dataset serves as the foundation for the integrative network-based and pathway enrichment analyses presented in the following sections, enabling a system-level understanding of apigenin’s mechanism of action in GI malignancies.

###  PPI Network Topology of Apigenin-Targeted Genes Across GI Malignancies

**Figure 1:**
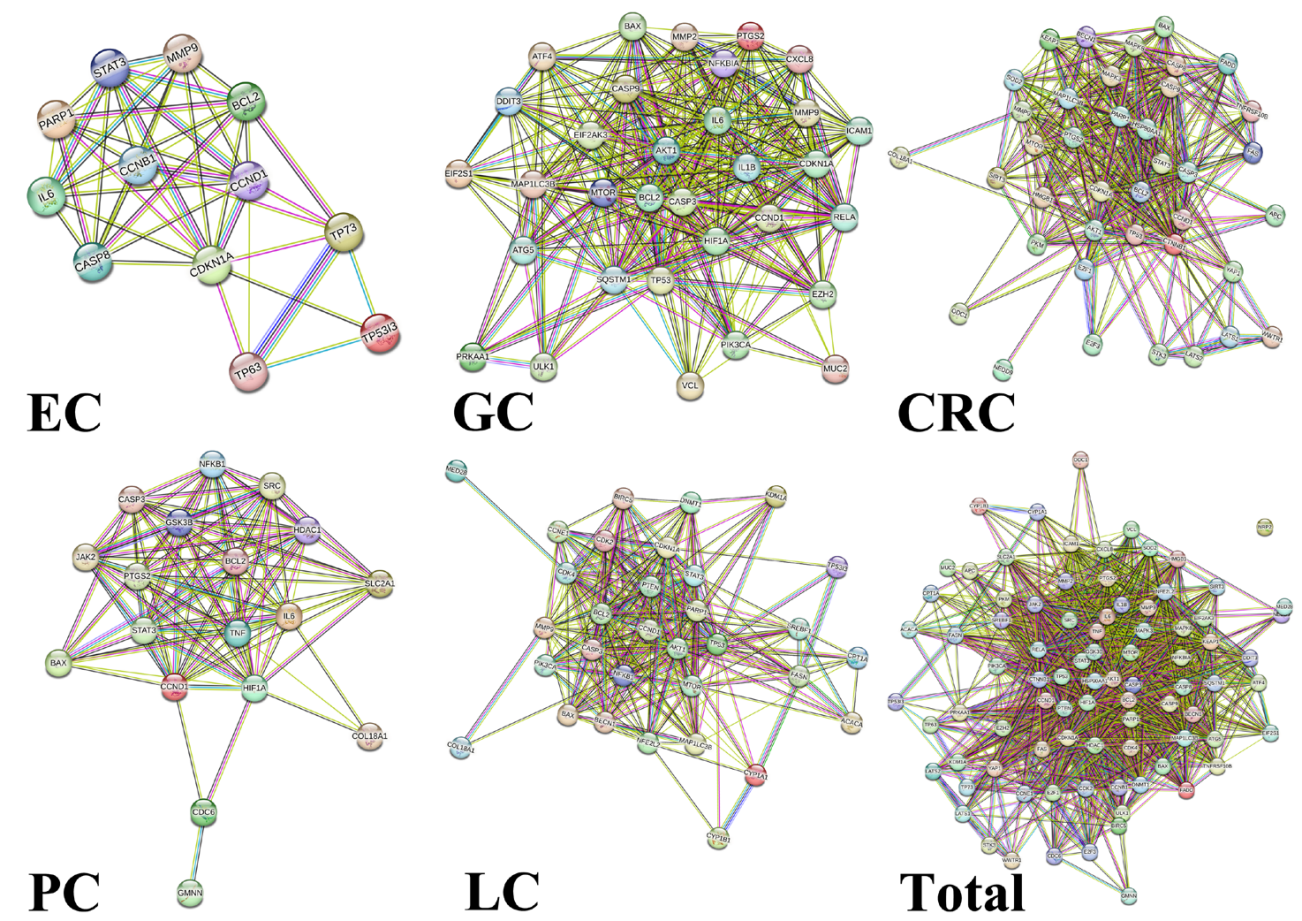
STRING-based protein–protein interaction (PPI) networks of apigenin-modulated genes in five GI cancers (EC, GC, CRC, LC, and PC) and an integrated meta-network. Networks were generated using a confidence score cut-off of 0.4. Higher interconnectivity indicates a stronger functional association among the target genes

 STRING-derived protein–protein interaction (PPI) networks revealed a complex interaction landscape of apigenin-responsive genes across five major GI cancers: EC, GC, CRC, PC, and LC. Notably, network complexity and connectivity varied significantly among these cancers (Figure 1). GC, CRC, and LC exhibited highly interconnected networks, indicating extensive functional convergence and suggesting that apigenin’smolecular influence may extend across broad oncogenic regulatory circuits. In contrast, the EC and PC networks appeared more compartmentalized and modular, implying that apigenin’s effects in these tumors may target more discrete pathways or specific functional modules.

 The recurrently identified central hub nodes, including TP53, BCL2, IL6, and STAT3, are well-established regulators of tumorigenesis and cellular stress responses. Their prominence within the network underscores their potential roles as pivotal mediators of apigenin’santicancer effects. The integrated meta-network combining all five cancer types revealed a densely interconnected core enriched with signaling molecules that govern apoptosis, inflammation, and cell cycle control, further reinforcing the hypothesis that apigenin modulates conserved oncogenic hubs shared across GI cancers. Apigenin’s ability to stabilize wild-type p53,^36,84,85^ suppress IL6/STAT3 signaling,^49,86-88^ and inhibit AKT and MAPK pathways^83,89^ supports its broad activity against dysregulated oncogenic axes. Collectively, these network topologies highlight the pleiotropic and multi-targeted mode of action of apigenin, pinpointing critical nodes for future mechanistic studies and potential therapeutic interventions.

###  Functional Enrichment Analysis Reveals Key Cancer Pathways Modulated by Apigenin

**Figure 2:**
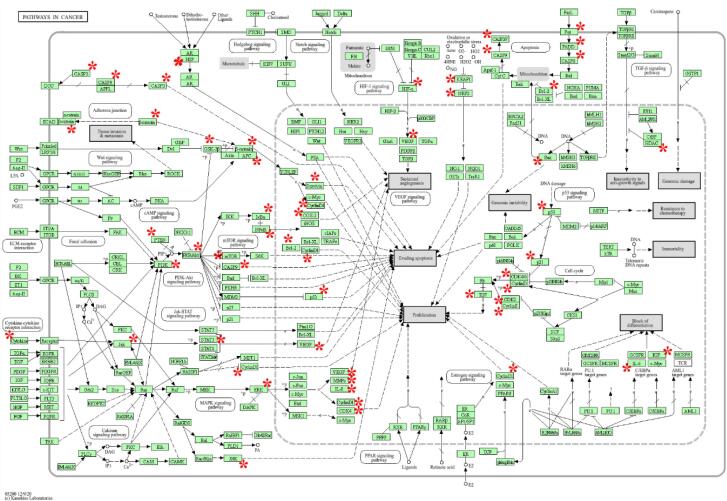
Schematic representation of major cancer-related pathways, highlighting the involvement of selected apigenin-responsive genes. The diagram was adapted from the KEGG “Pathways in Cancer” map (hsa05200), generated through DAVID functional annotation analysis. Modulated by apigenin are marked with red stars, illustratingtheir roles in proliferation, apoptosis, angiogenesis, genomic instability, and metastasis

 Functional annotation using KEGG via DAVID identified 29 apigenin-responsive genes associated with key cancer-related signaling pathways (Figure 2). Enrichment analyses prominently highlighted the PI3K-Akt,MAPK,JAK-STAT,mTOR,Wnt, and apoptosis pathways, with critical regulators such as AKT1, mTOR, MAPK1, STAT3, CCND1, and TP53 among the targets. Additionally, hallmark cancer-associated genes, including MYC, BCL2, CASP3, VEGFA, MMP2, and MMP9, were significantly modulated, demonstrating the broad impact of apigenin on oncogenic processes.

 Notably, this natural product also regulates genes involved in G2/M cell cycle arrest (e.g., cyclin B1 and geminin), DNA replication (e.g., CDC6), and apoptosis induction (e.g., CASP3), thereby disrupting proliferative signaling circuits.^72,73^ Moreover, several apigenin-responsive genes (particularly STAT3, AKT1, HIF1A, and IL6) are associated with poor prognosis and chemoresistance in GI cancers.^90^ Apigenin’s ability to downregulate STAT3 and AKT1 phosphorylation, suppress HIF1A, and modulate the tumor microenvironment provides a suggestive rationale for its integration into multi-agent therapeutic strategies.^91-93^ Given the pivotal roles of the PI3K-Akt-mTOR and Wnt/β-catenin pathways in tumorigenesis, chemoresistance, and metastasis in GI cancers.^1,94^ these findings suggest that apigenin exerts its anticancer effects by simultaneously modulating multiple key oncogenic signaling networks.

###  Gene Ontology Enrichment Analysis Supports Anti-Proliferative Mechanisms

**Figure 3:**
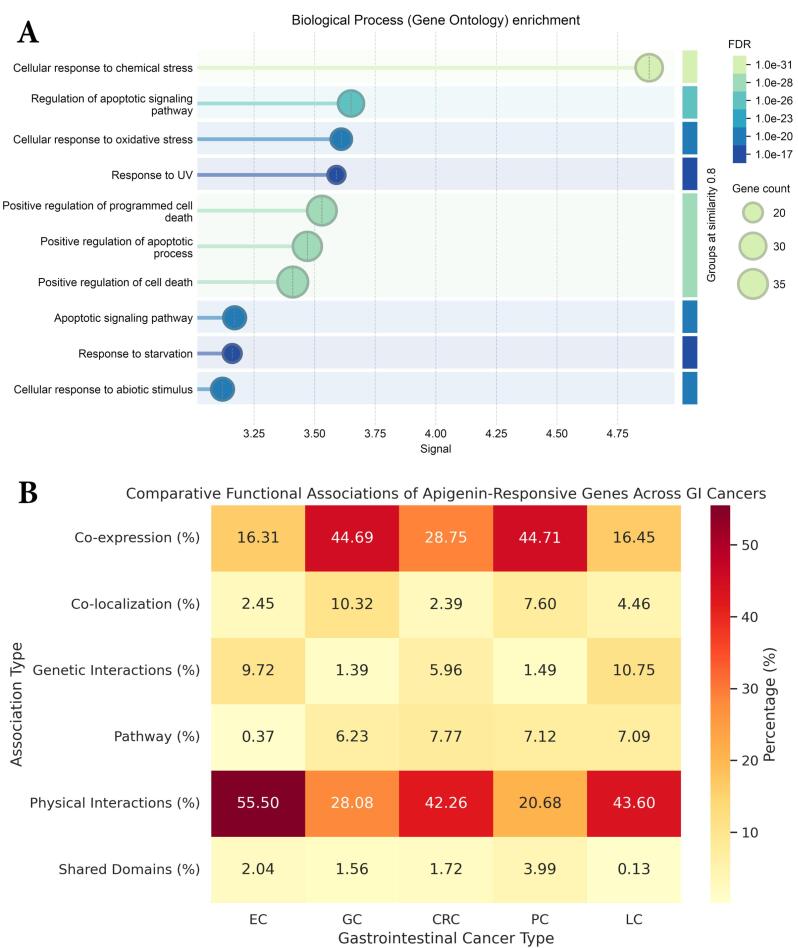
**(A)** GO Biological Process enrichment analysis of the selected genes using the STRING database. The most significantly enriched biological processes (GO terms) are displayed based on the input gene sets. The size of each circle represents the number of involved genes, while the color intensity indicates statistical significance (false discovery rate, FDR). Notably, processes related to the mitotic cell cycle, cell division, and chromosome segregation were highly enriched, suggesting a central role for these genes in proliferative and cell cycle regulatory pathways. **(B)** Heatmap illustrating the distribution of functional gene–gene associations among apigenin-responsive genes across GI cancers. This heatmap displays the relative frequencies of six types of gene–gene functional associations (co-expression, co-localization, genetic interactions, shared pathway involvement, physical protein–protein interactions, and shared protein domains) among differentially expressed genes regulated by apigenin in esophageal cancer (EC), gastric cancer (GC), colorectal cancer (CRC), liver cancer (LC), and pancreatic cancer (PC). Color gradients represent the proportion of gene pairs exhibiting each association type within each cancer. These differential topologies indicate that the molecular effects of apigenin are modulated by distinct functional gene networks that vary according to tumor type, underscoring the importance of cancer-context specificity in its mechanism of action

 GO Biological Process enrichment analysis using the STRINGplatform revealed predominant functional themes related to cell cycle regulation, including the mitotic cell cycle, chromosome segregation, DNA replication, and spindle organization (FDR < 1e−30; Figure 3A). Up to 35 apigenin-responsive genes are involved in these mitosis-associated processes, particularly those governing the G2/M checkpoint and spindle dynamics, consistent with apigenin’s well-established antiproliferative activity.^85,88^ These findings suggest that disruption of mitotic machinery and genome stability may underlie the therapeutic effects of apigenin, especially in liver and pancreatic cancers.

###  Comparative Functional Association Highlights Context-Dependent Mechanisms

 A comparative analysis of six types of gene–gene associations (co-expression, co-localization, genetic interactions, pathway co-membership, physical interactions, and shared protein domains) across GI cancers (Figure 3B) revealed tumor-type-specific interaction patterns. GC exhibited the highest rates of co-expression and co-localization, indicating strong transcriptional and spatial coordination of apigenin targets. In contrast, EC showed the lowest values, suggesting more dispersed gene activity. LC displayed the highest frequency of genetic interactions, while CRC genes were most frequently involved in shared pathway modules.

###  Network-Based Identification of Hub Genes Underlying the Actions of Apigenin

 Using degree centrality metrics in CytoHubba, we identified key hub genes within the PPI networks of each GI cancer (Table 4). Recurrently identified hubs (TP53, AKT1, CASP3, BCL2, and STAT3) highlighted core regulators of apoptosis, the cell cycle, and inflammation. Additional hubs, including CDKN1A, HIF1A, CCND1, IL6, and TNF, demonstrated cancer-specific prominence, indicating context-dependent regulatory roles. Integration across all cancers prioritized IL6, HIF1A, mTOR, TNF, and PTEN as universal therapeutic targets.

**Table 4:** Top-ranked hub genes in GI cancers. The top five hub genes for each GI cancer type were identified using degree centrality scores obtained from CytoHubba in Cytoscape. The “Total” column lists the top ten hub genes across all cancer types combined, based on integrated degree centrality metrics.

**EC**	**GC**	**CRC**	**PC**	**LC**	**Total**	
CDKN1A	TP53	TP53	HIF1A	TP53	AKT1	IL6
CCND1	BCL2	AKT1	IL6	AKT1	TP53	HIF1A
BCL2	CASP3	BCL2	TNF	PTEN	CASP3	mTOR
CCNB1	HIF1A	CASP3	CCND1	STAT3	BCL2	TNF
STAT3	AKT1	STAT3	GSK3B	CASP3	STAT3	PTEN

 These findings align with the established oncogenic signaling and inflammatory pathways targeted by apigenin,^83,86^ highlighting a conserved core of molecular regulators mediating its anti-gastrointestinal cancer effects.

###  Prognostic Implications of Apigenin-Responsive Genes

**Figure 4:**
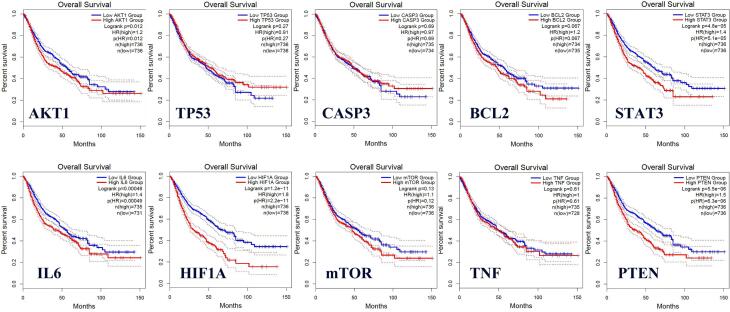
Kaplan–Meier overall survival curves for GI cancer patients stratified by high versus low expression of selected apigenin-responsive genes (AKT1, TP53, CASP3, BCL2, STAT3, IL6, HIF1A, mTOR, TNF, and PTEN), generated using the GEPIA platform. Significant associations were observed for HIF1A, STAT3, IL6, AKT1, and PTEN (*log-rank P < 0.05*)

 Kaplan–Meier survival analysis revealed significant associations between the expression of key hub genes and patient prognosis (Figure 4). Elevated expression of HIF1A, STAT3, IL6, AKT1, and PTEN was correlated with poorer overall survival across GI cancers, with HIF1A demonstrating the strongest prognostic significance (hazard ratio = 1.8). These findings underscore hypoxia- and inflammation-related pathways as critical drivers of tumor aggressiveness and potential mechanisms of treatment resistance.

 Interestingly, classical tumor suppressors and apoptosis regulators, such as TP53, CASP3, mTOR, and TNF, showed no significant associations with survival, suggesting that their roles may be more complex or context-dependent in these cancers. These prognostic markers warrant further validation in both experimental and clinical settings to refine apigenin-based therapeutic strategies.

###  Transcriptomic Landscape of Apigenin Targets

**Figure 5:**
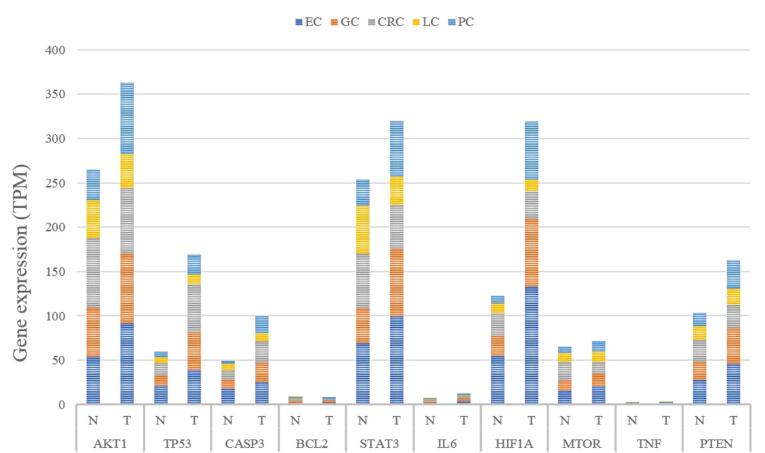
Stacked bar plot illustrating TPM-based gene expression levels in normal (N) and tumor (T) tissues across five GI cancer types: esophageal cancer (EC), gastric cancer (GC), colorectal cancer (CRC), liver cancer (LC), and pancreatic cancer (PC). The plot compares the expression patterns of ten key genes (AKT1, TP53, CASP3, BCL2, STAT3, IL6, HIF1A, mTOR, TNF, and PTEN) using data from TCGA and GTEx. Each bar represents the cumulative gene expression for each condition within the respective cancer type

 Analysis of transcriptomic expression (TPM values) across normal and tumor tissues in five GI cancers (Figure 5) revealed consistent upregulation of AKT1, STAT3, and HIF1A in tumors, reinforcing their roles as oncogenic drivers. Moderate expression differences in TP53 suggest possible compensatory transcriptional responses to the frequent loss-of-function mutations observed in GI tumors.

 The apoptosis-related genes CASP3 and BCL2 exhibited mild upregulation, indicating disrupted apoptotic homeostasis, while mTOR and PTEN showed moderate changes consistent with altered growth and metabolic signaling. The low transcript abundance of pro-inflammatory cytokines IL6 and TNF likely reflects their predominant expression in immune or stromal compartments rather than in tumor cells. This transcriptomic signature underscores the functional and prognostic significance of apigenin’s molecular targets, providing a basis for future proteomic and functional validation studies.

 On the other hand, combination strategies involving apigenin have demonstrated significant potential to enhance the efficacy of conventional chemotherapeutic agents across GI malignancies. In pancreatic cancer models, co-administration of apigenin with gemcitabine significantly improved anti-tumor activity by suppressing NF-κB signaling and inhibiting Akt activation, resulting in pronounced apoptosis and growth arrest compared to monotherapy.^95^ In colorectal cancer, apigenin has been shown to synergistically potentiate the effects of irinotecan by increasing CD26 expression approximately fourfold and to sensitizing tumor cells to TRAIL-induced apoptosis via upregulation of DR5 and activation of caspase pathways.^96^ These findings underscore the versatile role of apigenin in overcoming chemoresistance by modulating key survival and apoptotic pathways.

 Beyond colorectal and pancreatic contexts, apigenin demonstrates promising adjuvant properties in other GI cancer types. For instance, it mitigates inflammatory and oxidative stress in *Helicobacter pylori*-induced gastric carcinogenesis by inhibiting NF-κB and upregulating IκBα, thereby potentially preventing malignant progression.^97^ In cholangiocarcinoma models, apigenin induces robust apoptosis and tumor suppression by downregulating hnRNP H and A2/B1. Additionally, its broader mechanisms including modulation of PI3K/Akt/mTOR, Wnt/β-catenin, MAPK, and Nrf2 pathways, along with caspase activation and p53 upregulation provide a molecular basis for its chemosensitizing and chemoprotective effects across GI cancers. Hence, apigenin emerges as a multifaceted compound capable of enhancing the therapeutic index of standard chemotherapy regimens.^17,98^ Nevertheless, despite these encouraging preclinical findings, limitations such as poor bioavailability and the lack of large-scale clinical validation must be acknowledged, underscoring the need for well-designed clinical trials to confirm its translational relevance.

###  Binding Affinity Profiling of Apigenin Against Key Gastrointestinal Cancer Proteins

**Figure 6:**
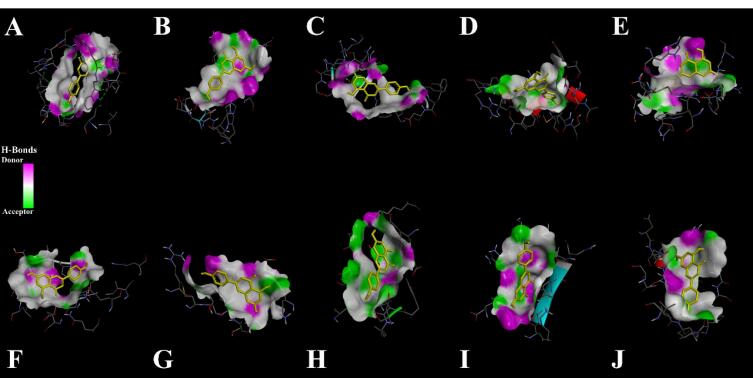
Molecular docking poses of apigenin with ten key target proteins, visualized using BIOVIA Discovery Studio. Apigenin is depicted in yellow stick representation, docked into the active sites of the target proteins (A–J). The protein surface is color-coded to highlight hydrogen bond donors (magenta), acceptors (green), and neutral/hydrophobic regions (gray), illustrating spatial complementarity and potential interaction pockets

 To elucidate the molecular interactions between apigenin and key proteins involved in gastrointestinal (GI) cancer signaling, molecular docking simulations were conducted on ten selected protein targets (Figure 6 and Table 5). Among these, AKT1 demonstrated the strongest binding affinity, with a docking score of –9.602 kcal/mol and an estimated inhibition constant (Ki) of 0.09 μM, indicating a highly potent and specific interaction. This high affinity is further supported by the formation of hydrogen bonds with critical residues in the ATP-binding pocket of AKT1, including Ser205, Asp292, and Gln79, which are crucial for the catalytic activity of the kinase. These interactions suggest that apigenin may inhibit AKT1 by directly competing with ATP binding or inducing conformational changes that impede kinase function.

**Table 5:** Molecular docking results of apigenin with selected GI cancer-related protein targets. The table presents the binding affinity (S-score, kcal/mol), estimated inhibition constant (Ki, μM), and identified hydrogen bond interactions for each protein.

**Targets**	**PDB ID**	**s-score**^a^ **(kcal/mol)**	**Ki**^b^ **(μM)**	**H-bond interactions**
AKT1	4EJN	-9.602	0.09	Ser-205, Asp-292, Gln-79
TP53	2AC0	5.968	2.37 × 10^10^	Asn-239
CASP3	1RHJ	-6.263	25.63	Arg-341
BCL2	4MAN	-7.21	5.18	-
STAT3	6NJS	-6.197	28.65	Thr-515, Lys-574, Asp-566
IL6	4NI9	-6.216	27.74	Pro-141
HIF1A	4ZPR	-6.385	20.86	Ser-113, Lys-125,
mTOR	4JSV	-6.875	9.12	Asp-2357, Asp-2191, Glu-2190
TNF	1TNF	-6.586	14.86	Ala-134, Asn-46, Gln-47
PTEN	1D5R	-7.188	5.38	Arg-173, Pro-169

^a^s-score: binding free energy ^b^Ki = e^ΔG/RT^, *R*= 1.986 cal/mol K, *T*= 298 K

 PTEN, a well-characterized tumor suppressor and regulator of the PI3K/AKT pathway, demonstrated considerable binding affinity (s-score = –7.188 kcal/mol; Ki = 5.38 μM), reinforcing its potential role in mediating the anti-cancer effects of apigenin. Other proteins, including BCL2 and mTOR, exhibited moderate but meaningful binding affinities (Ki = 5.18 μM and 9.12 μM, respectively), implicating these targets in apoptosis regulation and metabolic signaling pathways influenced by apigenin. In contrast, TP53 showed a positive binding energy (s-score = + 5.968 kcal/mol) and an exceptionally high Ki (2.37 × 10^10^ μM), indicating an unlikely direct interaction, possibly due to structural incompatibilities or steric hindrance within the active site.

 Recent evidence indicates that the effects of apigenin on TP53 are primarily mediated through indirect, signaling-dependent mechanisms rather than direct binding. For example, studies have demonstrated that apigenin induces apoptosis in human plasma cell lines by modulating the TP53 and STAT3 pathways, highlighting a complex interplay between these signaling networks in regulating cell death.^86^ Furthermore, research has shown that apigenin regulates the PI3K/AKT/p53 axis in breast cancer stem cells, leading to upregulation of TP53 expression and suppression of stemness features.^99^ Additional targets, such as CASP3, STAT3, IL6, HIF1A, and TNF, exhibit moderate binding affinities, with Ki values ranging from approximately 14 to 28 μM. These findings suggest that apigenin may modulate immune response, inflammation, and hypoxia pathways to some extent, although with less binding strength than AKT1 and PTEN. Collectively, these docking results highlight the selective binding profile of apigenin, primarily targeting pivotal nodes within the PI3K/AKT, apoptotic, and growth signaling pathways. This supports the concept of apigenin as a multi-targeted agent in GI cancers through the modulation of central oncogenic and tumor-suppressive proteins. Given that the reported Ki values are based on docking-predicted binding free energies under idealized conditions, these results should be interpreted with caution and considered approximate estimates of binding affinity.

 It should be noted that before docking apigenin, re-docking of known substrates for each target was performed to validate the docking protocol. The RMSD values ( ≤ 1.5 Å) and predicted binding free energies confirmed that the protocol reliably reproduced the experimental X-ray structures, ensuring that subsequent docking results are both robust and reproducible. The corresponding figure and table are provided in the Supplementary Information (Figure S1 and Table S1).

 In addition, molecular re-docking visualizations were generated using Discovery Studio 4.5 (Biovia, San Diego, CA). The comparison between the docked poses and the reference crystallographic conformations revealed a strong structural overlap, confirming the robustness of the docking procedure (Figure S1). Collectively, these results indicate that the re-docking process not only accurately reproduces ligand positioning within the active site but also preserves key intermolecular interactions. Therefore, the applied docking protocol can be considered highly reliable for the subsequent stages of this study.

###  Mechanistic Insights from Molecular Docking

 Molecular docking not only provided quantitative binding affinities but also offered mechanistic insights into the nature of apigenin-protein interactions. The strong affinity between apigenin and AKT1 aligns with previous studies, highlighting AKT1’s central role in the PI3K/AKT/mTOR signaling pathway, which is frequently dysregulated in GI malignancies.^34^ The hydrogen bonding network observed between apigenin and key ATP-binding residues suggests that apigenin effectively inhibits AKT1 kinase activity, potentially disrupting downstream proliferative and survival signaling.

**Figure 7:**
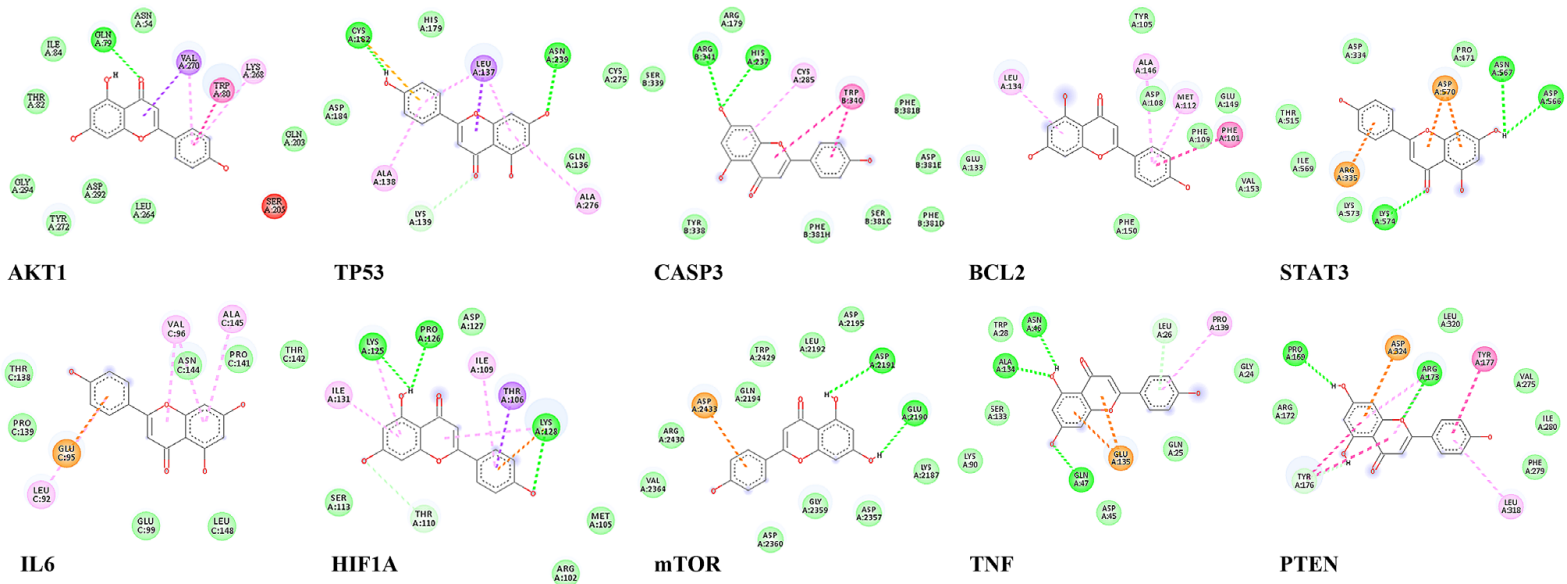
Two-dimensional schematic representations of molecular interactions between apigenin and ten selected hub proteins, generated using the BIOVIA Discovery Studio Visualizer. Each panel illustrates the binding interactions between apigenin and the residues of the target proteins, including van der Waals (light green), conventional hydrogen bonds (bright green), Pi-anion (orange), Pi-donor hydrogen bonds (pale green), Pi–Pi stacked (pink), Pi–Pi T-shaped (deep pink), and Pi–alkyl (light purple) interactions

 Conversely, the weak binding affinity of apigenin for TP53 suggests that its influence on this pivotal tumor suppressor is likely indirect, possibly mediated through the modulation of upstream regulators or downstream effectors rather than through direct interaction. Furthermore, two-dimensional interaction maps generated using BIOVIA Discovery Studio Visualizer (Figure 7) illustrate the binding modes of apigenin with mTOR, PTEN, STAT3, and other proteins, highlighting critical hydrogen bonds and hydrophobic contacts that stabilize these complexes. These detailed molecular insights complement the three-dimensional docking data and underscore the importance of non-covalent interactions in the multi-targeted anticancer activity of apigenin.

 In summary, combined docking analyses confirmed that apigenin exhibits selective, high-affinity interactions with key oncogenic proteins, particularly within the PI3K/AKT pathway. This provides a plausible molecular basis for its anti-cancer effects in GI tumors. These findings pave the way for further *in vitro* and *in vivo* studies to validate the therapeutic potential of apigenin as a multifaceted modulator of cancer signaling pathways.

 However, our comprehensive systems pharmacology approach delineates a multifaceted molecular framework underlying the anticancer effects of apigenin across diverse GI malignancies. The convergence on conserved signaling hubs and pathways (principally involving cell cycle control, apoptosis, inflammation, and hypoxia responses) illustrates the potential of apigenin as a pleiotropic agent capable of modulating core oncogenic networks. Moreover, tumor-specific network architectures and gene interaction patterns emphasize the necessity for context-aware therapeutic strategies.

 The identified hub genes (TP53, AKT1, STAT3, IL6, and HIF1A) serve as promising candidates for mechanistic studies and biomarker development, while their prognostic associations highlight their clinical relevance. Future experimental validation, including targeted functional perturbations and *in vivo* studies, will be critical to translating these findings into precision pharmacological interventions that exploit the anti-GI cancer potential of apigenin.

## Conclusion

 In summary, this comprehensive analysis delineates the multifaceted molecular landscape underlying the therapeutic potential of apigenin in major GI cancers. The identification of natural plant sources and their bioactive components provides a solid pharmacognostic foundation for the targeted extraction and formulation of apigenin-enriched agents. Integrative network analyses reveal that apigenin exerts pleiotropic anticancer effects by modulating key oncogenic hubs, such as TP53, AKT1, STAT3, BCL2, and HIF1A, which regulate critical cellular processes including apoptosis, cell cycle progression, and inflammatory signaling. The enrichment of apigenin-responsive genes in pivotal cancer-related pathways (such as PI3K-Akt, MAPK, JAK-STAT, and mTOR) highlights its capacity to simultaneously interfere with multiple tumorigenic mechanisms, potentially overcoming resistance pathways and limiting metastatic progression. Furthermore, the prognostic significance of several hub genes, notably HIF1A, IL6, and STAT3, underscores the clinical relevance of apigenin’s modulatory effects on hypoxia- and inflammation-driven tumor biology. Molecular docking studies complement these findings by demonstrating strong binding affinities of apigenin to critical signaling proteins, such as AKT1 and PTEN, suggesting a direct mechanistic basis for its anticancer activity. Collectively, these multi-level insights establish apigenin as a promising multi-targeted agent with broad-spectrum efficacy against GI malignancies. It should be noted that, despite apigenin’s poor aqueous and lipid solubility limiting absorption, its moderate lipophilicity, low molecular weight, and low polar surface area facilitate passive diffusion across membranes, including the blood–brain barrier. While apigenin can exert neuromodulatory effects and support barrier integrity via tight junction modulation, its oral bioavailability is constrained by rapid hepatic metabolism, which must be considered when designing *in vivo* studies or improving formulations for therapeutic use. Future, experimental validation and mechanistic studies are warranted to fully elucidate its therapeutic potential and optimize apigenin-based interventions in precision oncology.

## Competing Interests

 The authors declare no conflict of interest.

## Ethical Approval

 This study employed systems pharmacology approaches and did not involve direct experimentation on human participants or animals. The study protocol was reviewed and approved by the Ethics Committee of Sabzevar University of Medical Sciences (approval number: IR.MEDSAB.REC.1404.001). All analyses were conducted in accordance with relevant institutional and national ethical guidelines.

## Supplementary File

Supplementary file contains Figures S1 and Tables S1.
