# Supplementary material for: Apigenin as a Multi-Targeted Agent in Gastrointestinal Cancers: A Systems Pharmacology Approach
Source: Adv Pharm Bull. 2025 Oct 11;16(1):74–90. doi: 10.34172/apb.025.45970 (PMC13408527; doi:10.34172/apb.025.45970)
Supplement: Supplementary file 1 — Supplementary file contains Figures S1 and Tables S1. [file apb-16-74-s001.pdf]

Adv Pharm Bull 2026;16(1):74-S1
doi:10.34172/apb.025.45970
https://apb.tbzmed.ac.ir

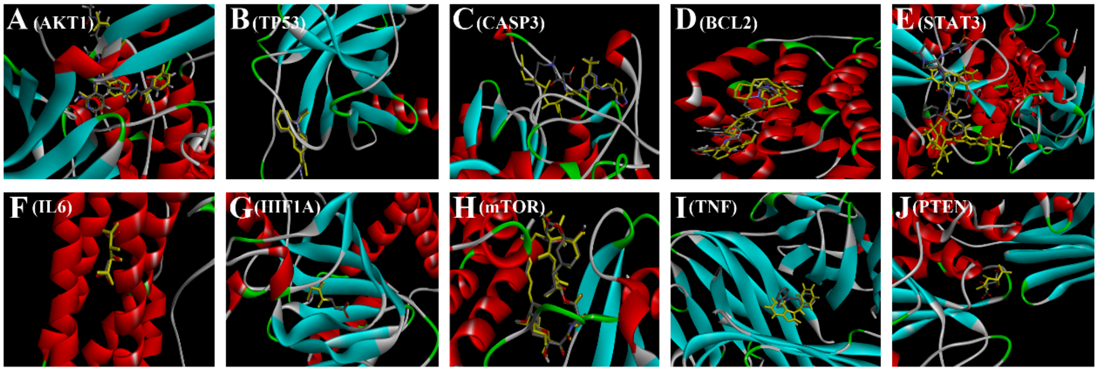

Fig. S1 Comparisons between different crystallographic structures (shown in gray) and re-docking data (shown in yellow). Relevant details are presented in Table 6.

Table S1. RMSD and re-docking scores for different ligand–protein complexes

**PDB ID**	4EJN	2VUK	1RHJ	4MAN	6NJS	1ALU	5l9b	4JSV	6X83	1D5R
**Target**	AKT1	TP53	CASP3	BCL2	STAT3	IL6	HIF1A	mTOR	TNF	PTEN
**Ligand**	N-(4-{5-[3-(acetylamino)phenyl]-2-(2-aminopyridin-3-yl)-3H-imidazo[4,5-b]pyridin-3-yl}benzyl)-3-fluorob	1-(9-ethyl-9H-carbazol-3-yl)-N-methylmethanamine	3-(2-{5-TERT-BUTYL-3-[(4-METHYL-FURAZAN-3-YLMETHYL)-AMINO]-2-OXO-2H-PYRAZIN-1-YL}-	4-[4-({4'-chloro-3-[2-(dimethylamino)ethoxy]biphenyl-2-yl}methyl)piperazin-1-yl]-2-(1H-indol-5-	[(2-{[(5S,8S,10aR)-3-acetyl-8-({(2S)-5-amino-1-[(diphenylmethyl)amino]-1,5-dioxopentan-2-yl}carb	L(+)-TARTARIC ACID	2-OXOGLUTARIC ACID	ADENOSINE-5'-DIPHOSPHATE	1-benzyl-1H-benzimidazole	L(+)-TARTARIC ACID

	enzamide		BUTYRYLAMINO)-5-(HEXYL-METHYL-AMINO)-4-OXO-PENTANOIC ACID ANION	yloxy)-N-({3-nitro-4-[(tetrahydro-2H-pyran-4-ylmethyl)amino]phenyl}sulfonyl)benzamide	amoyl)-6-oxodecahydropyrrolo[1,2-a][1,5]diazocin-5-yl]carbamoyl}-1H-indol-5-yl)(difluoro)methyl]phosphonic acid (non-preferred name)					
**RMSD value**(A°)	1.12	0.64	0.76	0.91	1.26	0.43	0.66	0.89	0.87	0.72
**s-score**[a] (kcal/mol)	-7.74	-9.45	-9.77	-8.23	-7.96	-10.54	-9.31	-8.98	-9.05	-10.11
**Ki**[b] (μM)	2.10	0.117	0.068	0.92	1.45	0.019	0.138	0.25	0.19	0.041

[a] S-score: binding free energy

[b] $Ki = e^{\Delta G/RT}$, R = 1.986 cal/mol K, T = 298 K
